# Drs. Allen and Hunter,—Priority of Improvement

**Published:** 1853-01

**Authors:** 


### DRS. ALLEN AND HUNTER,—PRIORITY OF IMPROVEMENT.

It will be seen by Dr. Allen's advertisement that he is determined to defend his patent; and hence has instituted suit against Dr. Hunter. It can hardly be expected that the Profession generally will take the same interest in the suit which these individuals may. We are not much acquainted with the patent laws, having never visited the *great* office, or held a *correspondence* with any of the officers; but we suppose the suit will determine the validity of Dr. Allen's patent; and settle effectually the controversy which has existed for the last year or two.

